# Nutritional intervention for the prognosis of nasopharyngeal carcinoma chemoradiotherapy patients: A meta-analysis

**DOI:** 10.1097/MD.0000000000035386

**Published:** 2023-10-13

**Authors:** Ying He, Xiaoyu Chen, Tong Yang, Yan Li, Sitao Tan, Xiaoxia Liu

**Affiliations:** a Department of Pharmacy, Guangxi Academy of Medical Sciences and the People’s Hospital of Guangxi Zhuang Autonomous Region, Nanning, China; b Pharmaceutical Colleague, Guangxi Medical University, Qingxiu District, Nanning, China.

**Keywords:** chemoradiotherapy, meta-analysis, nasopharyngeal carcinoma, nutrition

## Abstract

**Background:**

To conduct a meta-analysis to investigate the effects of different nutritional interventions on various serum nutritional indicators and patients’ prognosis during radiotherapy for nasopharyngeal carcinoma, to assess treatment safety and efficacy.

**Methods:**

A systematic literature search, mainly randomized controlled trials (RCTs), on the effects of nutritional support on patients undergoing radiotherapy for nasopharyngeal carcinoma was conducted between January 2010 and August 2022 using databases such as China National Knowledge Infrastructure, Wanfang Database, Web of Science, PubMed, Cochrane Library, and Embase. Risk of bias was assessed using the Cochrane Rob2 scale. The meta-analysis was performed using Stata 17.0 software, and the heterogeneity between studies was assessed using the I^2^ test, and funnel plots were used to qualitatively assess publication bias.

**Results:**

Overall, 10 RCTs with a total sample size of 879 cases were identified. The meta-analysis results showed that body mass index (BMI) (odds ratio = 0.026, 95% confidence interval^[1]^: −0.348 to 0.401, *P* > .05), albumin (standardized mean difference [SMD] = 0.13, 95% CI: −0.127 to 0.387, *P* > .05), and total protein levels were not significantly different between the nasopharyngeal cancer (NPC) radiotherapy group with nutritional support group (SMD = −0.262, 95% CI: −1.062 to 0.537, *P* > .05) and the control group; pre-albumin (SMD = 0.256, 95% CI: 0.022–0.491, *P* = .032), hemoglobin (SMD = 0.436, 95% CI: 0.26–0.612, *P* < .000), and lymphocyte count (SMD = 1.125, 95% CI: 0.868–1.381, *P* < .000) were significantly higher in the nutritional intervention group than in the control group.

**Conclusion:**

Compared with conventional diets, nutritional interventions can improve serum nutritional parameters, nutritional status, treatment tolerance, and prognosis of patients undergoing radiotherapy for nasopharyngeal carcinoma.

## 1. Introduction

Nasopharyngeal cancer (NPC) is a common malignancy of the head and neck in southern China, Southeast Asian, and East Asian regions. Approximately 92% of newly diagnosed NPC cases occur in developing countries^[1]^, and the NPC incidence and associated mortality rates in China are higher than the global average, with NPC-associated deaths in China accounting for nearly 40% of the total deaths from NPC worldwide.^[2]^ Data from the National Cancer Center China reveal that China crude incidence rate of 2.7 in 1990 increased to 7.8 per 100,000 people in 2019 (increase of 188.9%), which is lower than that of developed countries in Europe and the United States, indicating a relatively high burden due to NPC in China.^[3,4]^

Radiotherapy is not effective for most patients with NPC. With current advances in diagnostic imaging and chemoradiotherapy, a combination of radiotherapy-based treatment methods together with concurrent chemotherapy has gradually becoming the standard of care for NPC.^[5]^

More than 50% of patients with a first clinical diagnosis of malignancy are also at varying degrees of nutritional risk.^[6]^ Because of the specific location of the NPC lesion, most patients with an NPC have a lesion that specifically causes compression of the nasopharynx and pharynx, followed by infiltration of the local digestive tract mucosa, invasion of nerves and peripheral blood vessels, and local inflammatory edema,^[7]^ all of which can cause clinical symptoms such as pain in the throat, nasal congestion, bleeding, dizziness and headache, and difficulty in swallowing, resulting in decreased appetite, reduced nutritional intake, and nutritional risk. During chemotherapy or radiotherapy treatment, most part of the chemotherapy drugs and radiation will cause damage to the mucous membrane of the lesion and induce adverse reactions such as oral microsites, which will affect the intake and absorption of nutrients.^[8,9]^ The occurrence of nutritional risks will, in turn, affect the outcome and prognosis of the patient.

Several epidemiologists have conducted studies on the effects of serological nutritional indicators in patients with cancer; for instance, serum prealbumin levels can be used to identify patients at risk of malnutrition. Malnutrition is a significantly weak prognostic factor.^[10–13]^ A prospective study showed that a low level of preoperative serum albumin in patients with cancer was indicative of poorer overall survival and an increased risk of postoperative complications, which could therefore be an independent prognostic factor.^[14]^ In addition to body mass index (BMI) and prealbumin, albumin and total lymphocyte count are good predictors of the risk of postoperative complications; the smaller the albumin and total lymphocyte count values, the higher the subjective overall assessment score and the less favorable the survival outcome.^[15]^ Accumulating evidence suggests the positive effects of nutritional interventions. Nutritional status is closely related to the outcome and prognosis of patients treated with radiotherapy for NPC.^[16–19]^

Despite the number of studies related to nutritional interventions for NPC, there is a lack of evidence on the nutritional indicators that improve a patient condition before and after the nutritional interventions. Therefore, we conducted a systematic review and meta-analysis of randomized controlled trials (RCTs) to investigate the effects of different nutritional interventions on serum nutritional indicators and prognosis during radiotherapy for NPC to assess treatment safety and efficacy.

## 2. Materials and methods

### 2.1. Protocol and registration

In addition to the PRISMA guidelines, we followed the Cochrane Handbook for Systematic Reviews of Intervention.^[20]^ The protocol for this systematic review and meta-analysis was registered in PROSPERO (no.: CRD42022366089).

### 2.2. Search strategy

We performed a literature search in various databases such as China National Knowledge Infrastructure, Wanfang Database, China Science and Technology (Sci-Tech) Journal Database, PubMed, Cochrane Library, Embase, and Web of Science databases. The keywords used for the search included “nasopharyngeal carcinoma, nutrition, chemoradiotherapy, radiation nasopharyngeal carcinoma, nutrition, chemoradiotherapy, radiation,” “randomized controlled trial,” and “controlled clinical trial.” Literature searches were conducted using medical subject headings and synonyms. The detailed search strategy is available in Supplemental Digital Content (Table S1, http://links.lww.com/MD/K108). The search period spanned from January 2010 to August 2022.

### 2.3. Study eligibility criteria

Year of publication to August 2022.

The study type should have been an RCT on nutritional support.

The study population should have included patients with NPC, with radiotherapy as the final treatment type.

The interventions in the experimental group should have been clearly described.

The study should have passed ethical review.

Primary outcomes: Relevant nutritional indicators reported in the literature, such as BMI, albumin level, hemoglobin, body weight, prealbumin, lymphocyte count, and total protein.

Secondary outcomes: Oral mucositis.

### 2.4. Exclusion criteria

Duplication of the same literature in different databases.

Study population concurrently experienced other cachectic diseases.

Receipt of other drugs or treatments that may affect the observed nutritional indicators.

Literature with flaws in study design.

Literature for which complete data are not available or full text is not available.

Literature other than RCTs.

### 2.5. Selection of studies and data extraction

Literature search of the databases was conducted by 2 researchers (Yang and He), wherein they performed an initial screening of the titles and abstracts and removed duplicates to ensure that each article was screened and evaluated, and in case of disagreement between the 2 reviewers, a third researcher made the assessment. One researcher (Liu) extracted data from the retrieved literature, including information on primary and secondary outcome indicators. Then 2 researchers (He and Chen) cross-reviewed and collated the tables. They ensured that basic information about the article, such as literature title, authors’ details, year of publication, study population, intervention, and conclusion, was collected.

### 2.6. Risk of bias assessment

Methodological quality of the included studies, namely, RCTs, was assessed by 2 researchers (He and Yang), using the evaluation criteria provided by the Cochrane Literature Quality Assessment Tool, based on 7 aspects: method of generating the random allocation sequence, allocation concealment, blinding of the investigators and subjects, blinding of outcome evaluation, completeness of outcome data, selective reporting of study results, and other biases, which were used to rate the articles as “low risk, “some concerns, and “high risk.”^[21]^

### 2.7. Data analysis

The meta-analysis was performed using STATA 17.0. Systematic evaluation was performed through a meta-analysis of continuous variables (BMI, albumin, prealbumin, hemoglobin, lymphocyte count, and total albumin index) and dichotomous variables (incidence of adverse reactions). Data were analyzed using the STATA 17.0 statistical package (Cochrane Collaboration Software). Data of dichotomous outcomes were expressed as odds ratios with 95% confidence intervals (CIs) and standardized mean difference (SMD). A test of heterogeneity was performed with the I^2^ test and Q statistic. An I^2^ value of > 50% or *P* value of < .05 was assumed to indicate significant heterogeneity. Publication/reporting biases were visually assessed using funnel plots. If there was no observed heterogeneity, then the fixed-effects model was chosen; otherwise, the random-effects model was used.^[22,23]^

## 3. Results

### 3.1. Study identification and selection

**Figure 1.**
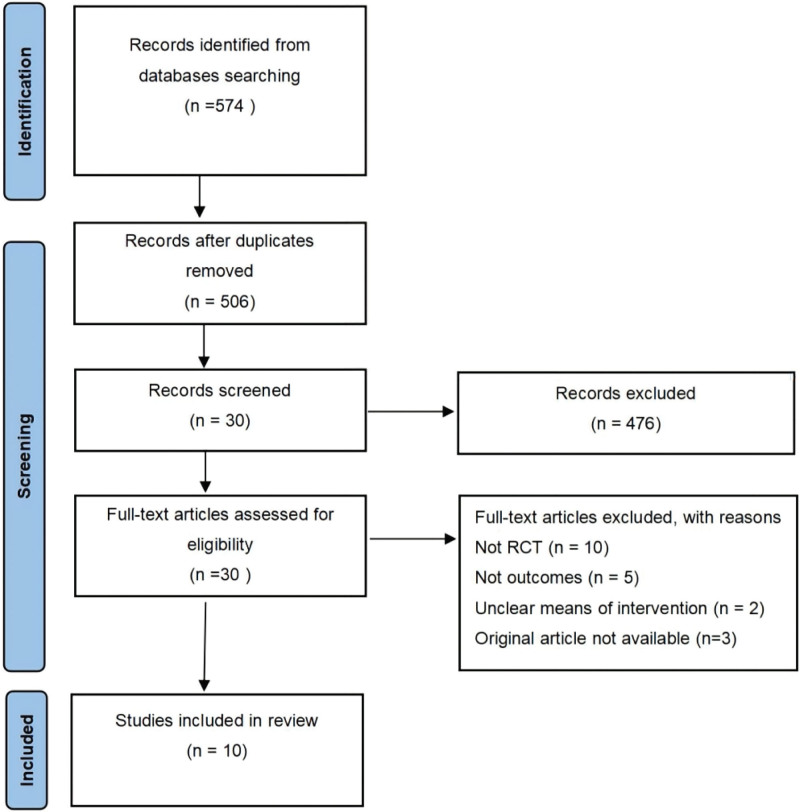
The process of selecting studies for inclusion in the meta-analysis.

The search strategy yielded a total of 574 records, wherein 68 duplicate studies were removed, 30 remained after initial screening and re-screening according to the inclusion and exclusion criteria, and 476 were excluded; the remaining literature was reevaluated to exclude articles that were not RCTs, those for which the original text was not available, those that did not describe outcome indicators, and those that had evaluated nonprimary lesions; altogether, only 10 studies (4 in English and 6 in Chinese) were included for analysis (Fig. 1). The total sample size of the included studies was 879 patients. Details of the 10 RCTs^[24–32]^ included in the meta-analysis are listed in Table 1.

**Table 1:** The characteristics of the included studies.

Author/year	Country	Study design	Sample size (exp/control)	Age of patients (yr)	Gender (male/female)	Intervention	Nutrition intervention description	Estimation of quality
Shengjin Dou/2022	China	RCT	42 (23/19)	E: 48 (18–63) C: 47 (34–63)	E: 20/3 C: 13/6	ONS	ONS (Methuselah Medical Technology) 20 g, tid	High risk of bias
Zekai Shu/2020	China	RCT	101 (67/34)	51 (18–73)	122/54	EN	NR	Low risk of bias
Shuang H/2020	China	RCT	114 (58/56)	E: 49.07 ± 9.16 C: 51.2 ± 7.89	E: 44/14 C: 39/17	ONS	ONS (Nutrison, Nutricia Pharmaceutical Co) 30 kcal/kg/d, 400 ml/d	Some concerns
Haizhen Y/2020	China	RCT	46 (25/21)	NR	E: 19/6 C: 16/5	EN	NR	Low risk of bias
Xueyan W/2020	China	RCT	90 (45/45)	NR	E: 29/16 C: 33/12	ONS	ONS (Nutrison, Nutricia Pharmaceutical Co) 25–30 kcal/kg	Some concerns
Wen J/2019	China	RCT	100 (50/50)	E: 46.7 ± 10.87 C: 48.18 ± 11.13	E: 33/17 C: 36/14	ONS	ONS (Niufutai, EnterNutr) 402 kcal/d	Low risk of bias
Yuanyuan C/2019	China	RCT	114 (58/56)	E: 49.07 ± 9.16 C: 50.98 ± 8.02	E: 44/14 C: 39/17	ONS	ONS (Nutrison, Milupa GmbH) 30 kcal/kg, 400–600 ml/d	Low risk of bias
Danhua D	China	RCT	80 (40/40)	E: 46.35 ± 9.43 C: 46.47 ± 9.32	E: 26/14 C: 25/15	ONS	ONS (Ensure, Abbott Pharmaceutical Co) 55 g/d, tid	Some concerns
Dongjing H	China	RCT	106 (55/51)	E: 56.6 C: 55.8	E: 47/8 C: 43/8	EN	EN preparation was prepared by the nutrition department	High risk of bias
Juan Z	China	RCT	86 (43/43)	E: 52.8 ± 9.1 C: 53.1 ± 8.8	E: 33/10 C: 34/9	EN	EN preparation was prepared by the nutrition department	Some concerns

EN = tube feeding enteral nutrition, ONS = oral nutritional supplements, RCT = randomized clinical trial.

**Figure 2.**
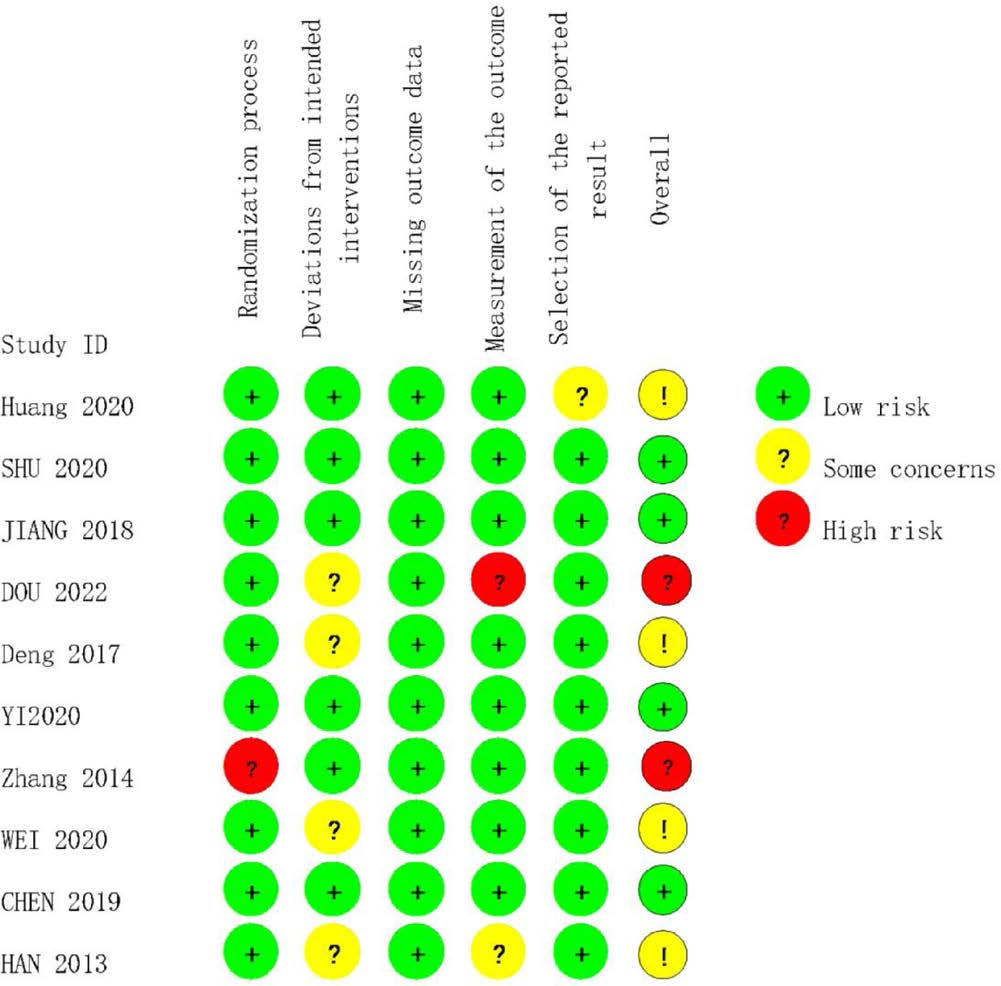
Quality assessment of Cochrane.

The risk of bias in the included studies was evaluated by 2 researchers (He and Yang), using the risk of bias tool ROB2.0 of the Cochrane Handbook for Systematic Evaluation of Interventions.^[21]^ The risk of bias was calculated by evaluating the following areas of bias: randomization process, deviation from established interventions, missing outcome data, outcome measures, selective reporting of outcomes, and overall bias assessment. Almost half of the RCTs had a low risk of bias,^[24,25,28,32]^ while almost half of the RCTs had some risk of bias owing to unclear allocation sequence concealment methods or the influence of the study setting,^[25,27,28,30]^ and 2 RCTs had a high risk of bias owing to problems with outcome measures.^[26,31]^ The results of the risk of bias assessment are presented in Figure 2.

### 3.2. Nutritional status of patients undergoing radiotherapy for NPC

#### 3.2.1. Changes in BMI before and after the intervention.

**Figure 3.**
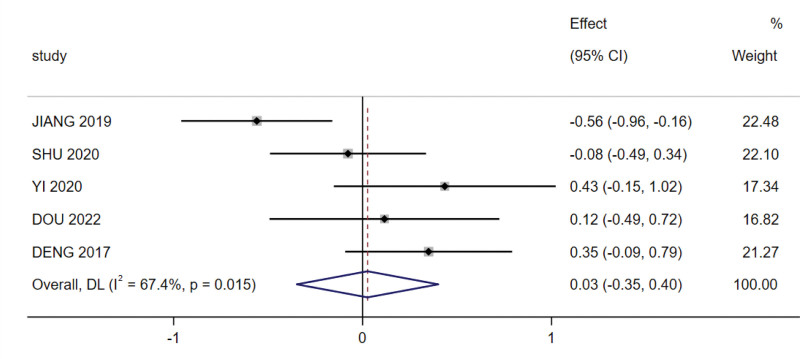
Nutrition indicators meta-analysis: body mass index (BMI).

Five studies (329 patients) analyzed the BMI of patients undergoing radiotherapy for NPC with nutritional support. The meta-analysis indicated that there was no significant difference in the value of change in BMI before and after radiotherapy (SMD = 0.026, 95% CI: −0.348 to 0.401, *P* = .891; Fig. 3), as determined using a random-effects model and that there was heterogeneity between studies (I^2^ = 67.4%, *P* = .015).

#### 3.2.2. Changes in blood markers.

**Figure 4.**
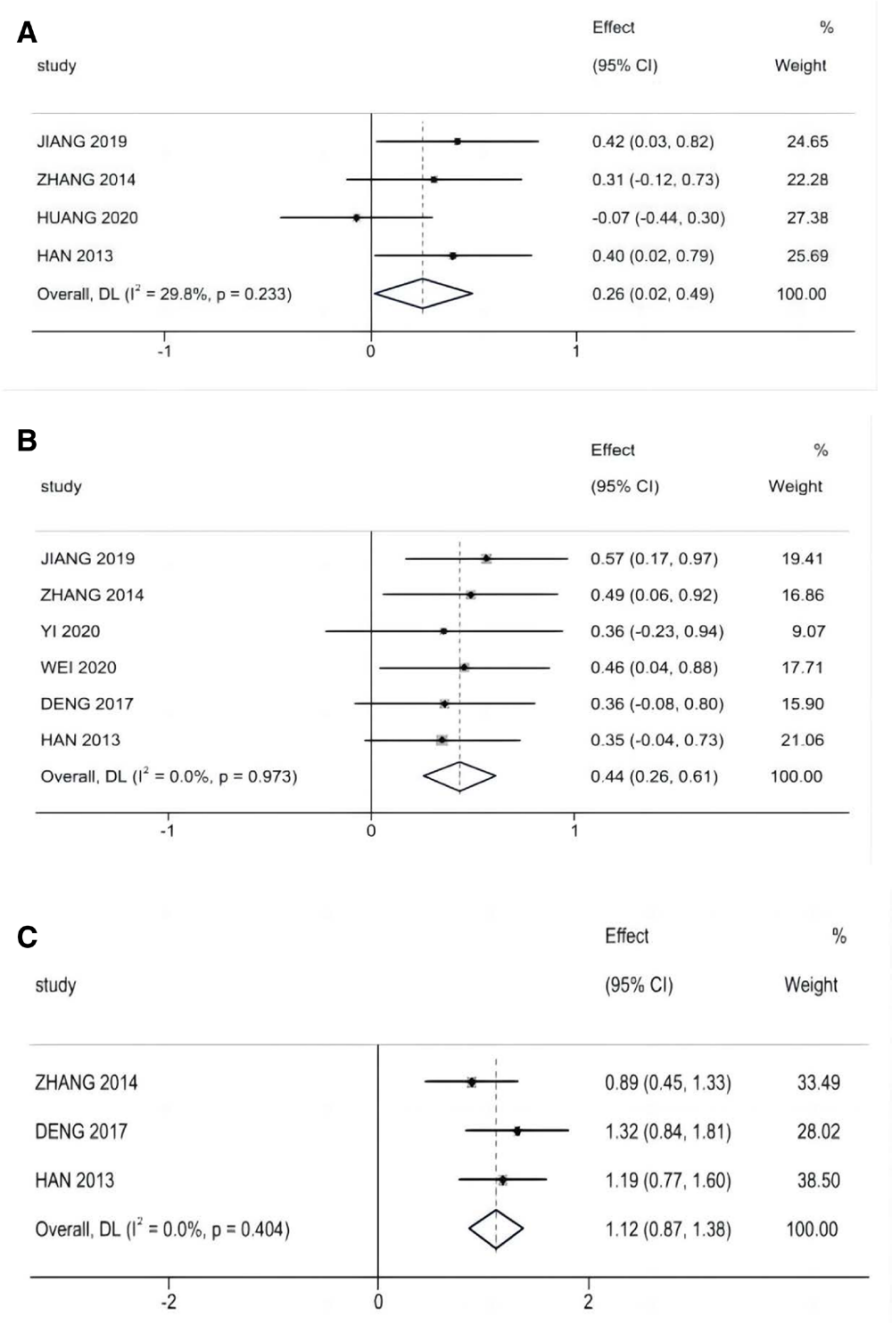
Nutrition indicators meta-analysis: (A) Pre-albumin, (B) Hemoglobin, (C) Lymphocytes.

For pre-albumin, hemoglobin, and lymphocyte count, 4 studies (406 patients), 6 studies (453 patients), and 3 studies (138 patients) analyzed the change in values before and after radiotherapy in patients with NPC receiving nutritional support (Fig. 4), all without heterogeneity (I^2^ = 29.8%, I^2^ = 0%), as determined using a fixed-effects model, compared with that in the control group. A significant difference was observed in the change in the reduction of the prealbumin value in patients undergoing radiotherapy for NPC when compared with that of the control group (SMD = 0.256, 95% CI: 0.022–0.491, *P* = .032); the hemoglobin levels in the experimental group of patients undergoing radiotherapy for NPC and receiving the nutritional intervention were increased, with a significant difference between the 2 groups (SMD = 0.436, 95% CI: 0.26–0.612, *P* < .001); lymphocyte counts were reduced after the nutritional intervention, with a significant difference between the experimental and control groups (SMD = 1.125, 95% CI: 0.868–1.381, *P* < .001).

**Figure 5.**
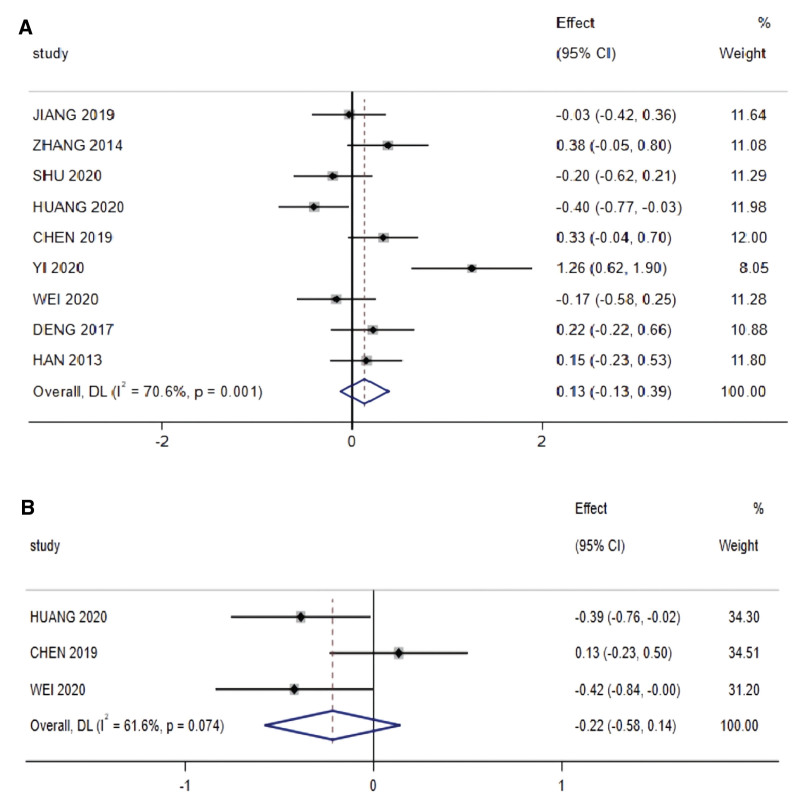
Nutrition indicators meta-analysis: (A) Albumin and (B) Total protein.

Among the included studies, 9 and 3 described albumin and total protein levels (Fig. 5), respectively, and the corresponding results of the heterogeneity test were I^2^ = 70.6% and I^2^ = 61.6%, both obtained using a random-effects model; no significant difference was observed in the change in reduced albumin levels in patients undergoing radiotherapy for NPC and receiving nutritional intervention when compared with the control group (SMD = 0.13, 95% CI: −0.127 to 0.387, *P* > .05); no significant difference was noted in the change in total protein values after nutritional intervention (SMD=−0.22, 95% CI: −0.58 to 0.14, *P* > .05).

#### 3.2.3. Adverse events.

**Figure 6.**
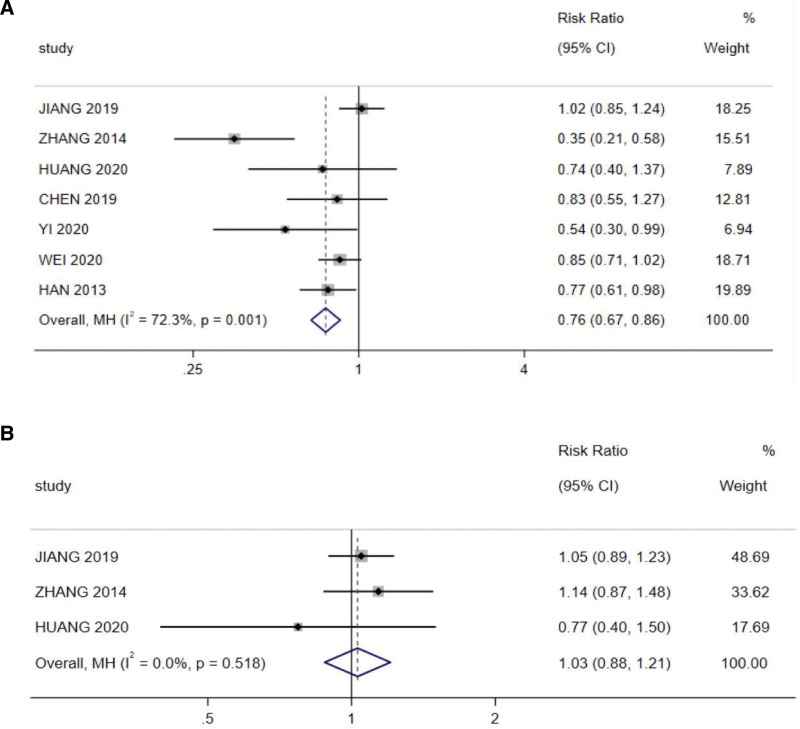
Adverse effects meta-analysis: (A) Oral mucositis and (B) Radiation dermatitis.

Oral mucositis are a serious adverse effect during radiotherapy for patients with NPC. Seven studies mentioned the incidence of oral microsites, and the meta-analysis showed a significant difference between the incidence of oral mucositis in the experimental and control groups (risk ratio = 0.75, 95% CI: 0.61–0.932, *P* < .001, I^2^ = 67.2%). However, different results were observed in terms of the adverse effect of radiation-associated dermatitis, the incidence of which was reported in 3 studies, and the analysis of this indicator showed no significant difference between the experimental group and the control group (risk ratio = 1.029, 95% CI: 0.878–1.206, *P* = .722, I^2^ = 0%; Fig. 6).

## 4. Subgroup analysis and meta-regression

The test for heterogeneity in this study was significant, indicating heterogeneity between the included studies’ outcomes. To analyze the sources of heterogeneity, subgroup analyses were conducted according to the mode of nutritional intervention and radiotherapy dose for NPC.

### 4.1. Nutrition interventions

In 6 studies, the nutritional intervention was oral supplementation, and in 4 studies, the intervention was individualized enteral nutrition, with no significant differences in any of the outcome indicators in the subgroups.

**Figure 7.**
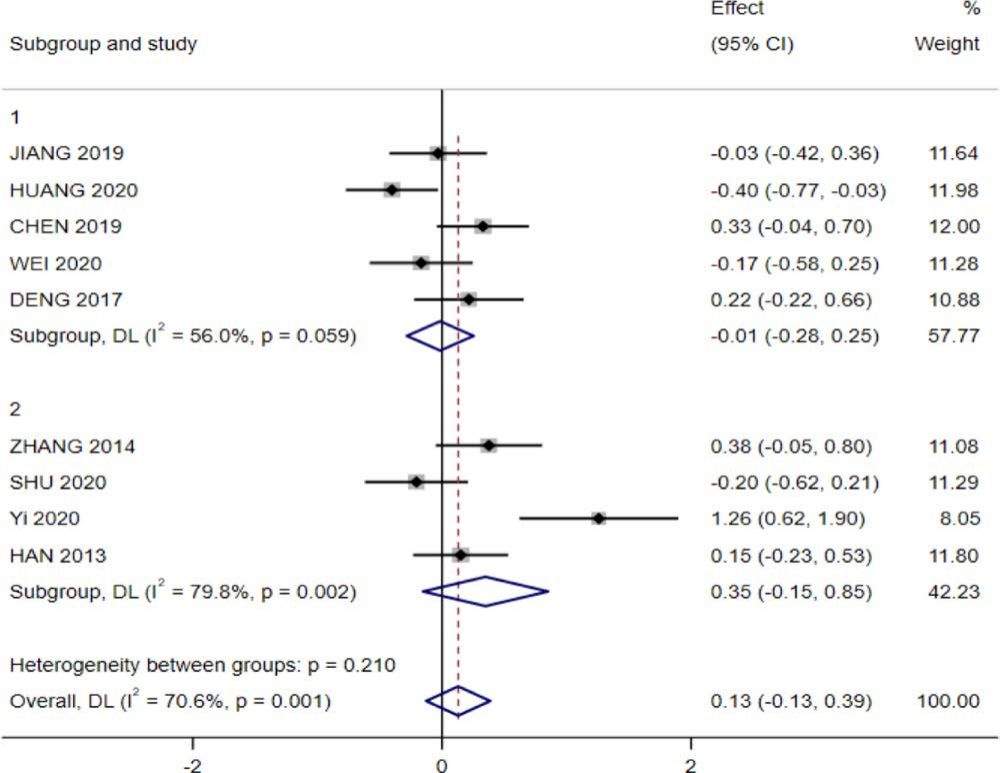
Subgroup analysis of the effect of nutritional intervention modalities on albumin in forest plots.

In a subgroup analysis examining the effect of different intervention modalities on albumin levels, 5 RCTs were included in the subgroup for oral nutritional supplementation and 4 RCTs in the subgroup for individualized enteral nutrition, with a large heterogeneity obtained between studies (*P* < .01, I^2^ = 70.6%) using a random-effects model. The meta-analysis results showed heterogeneity between subgroups for oral nutritional supplementation (I^2^ = 56%, *P* = .059), which was not significant (SMD = −0.111, 95% CI: −0.28 to 0.25, *P* = .916), and heterogeneity between subgroups for individualized enteral nutrition (I^2^ = 79.8%, *P* < .05), which was not significant (SMD = 0.35, 95% CI: −0.15 to 0.85, *P* = .172; Fig. 7).

**Figure 8.**
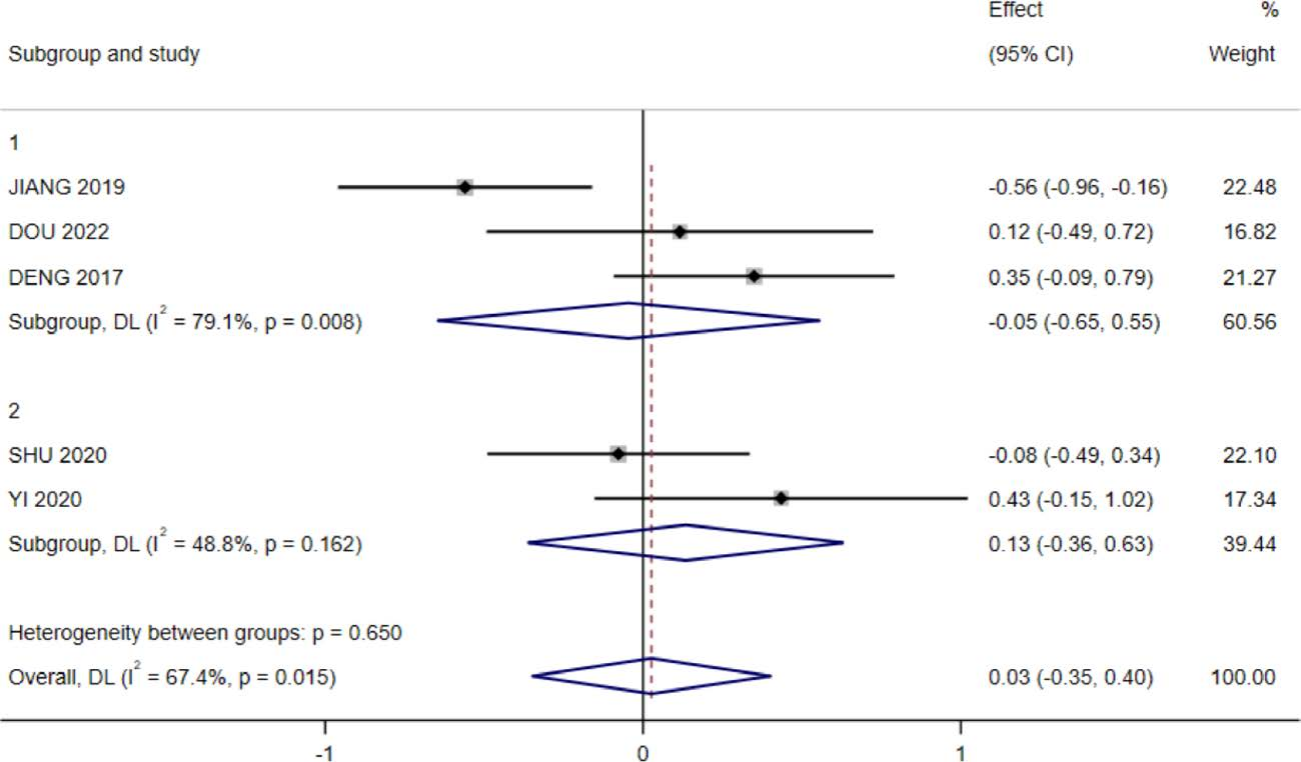
Subgroup analysis of the effect of nutritional intervention modalities on BMI forest plots. BMI = body mass index.

In a subgroup analysis examining the effect of different interventions on BMI, a to3tal of 3 RCTs were included in the subgroup of oral supplementation and 2 in the subgroup of individualized enteral nutrition, with high heterogeneity between studies (*P* < .01, I^2^ = 67.4%), as determined using a random-effects model, The meta-analysis results showed no effect on BMI in the subgroup of oral supplementation and high heterogeneity between studies (I^2^ = 79.1%, *P* = .008), with insignificant results (SMD = −0.045, 95% CI: −0.65 to 0.55, *P* = .882); the subgroup of individualized enteral nutrition had no effect on BMI, and the difference was not significant (SMD = 0.134, 95% CI: −0.348 to 0.401, *P* = .594; Fig. 8).

### 4.2. Radiotherapy dose

A consensus on NPC suggested that the toxic side effects of radiotherapy caused by the standard dose of 70 Gy are yet to be addressed. This analysis has been discussed in 2 subgroups based on radiotherapy dose.

**Figure 9.**
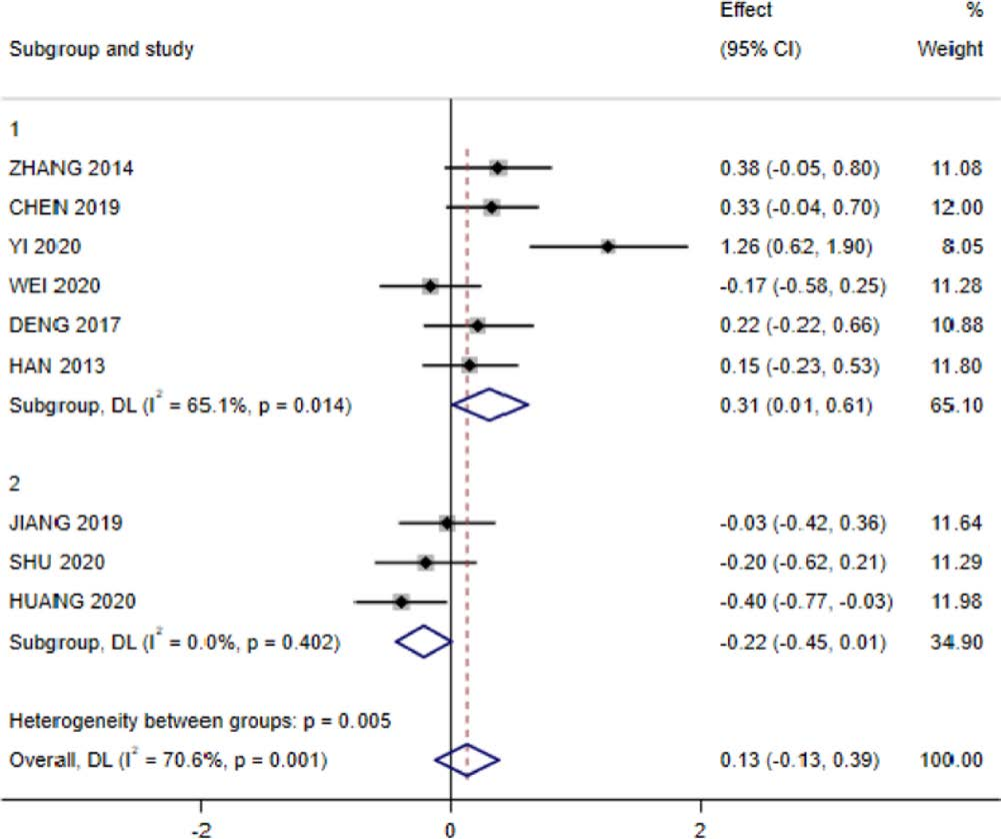
Forest plot for subgroup analysis of the effect of radiotherapy dose on albumin.

In subgroup analysis examining the effect of radiotherapy dose on albumin, 6 RCTs were included in the subgroup of ≥ 70 Gy and 3 RCTs in the subgroup of < 70 Gy. The analysis showed that heterogeneity existed between studies in the subgroup of ≥ 70 Gy (I^2^ = 65.1%; *P* = .014), with significant results (SMD = 0.311, 95% CI: 0.012–0.61; *P* < .05); no heterogeneity was observed in the subgroup of < 70 Gy (I^2^ = 0.00%; *P* = .402) and no significant difference (SMD = 0.13, 95% CI:−0.127 to 0.387; *P* = .056; Fig. 9).

**Figure 10.**
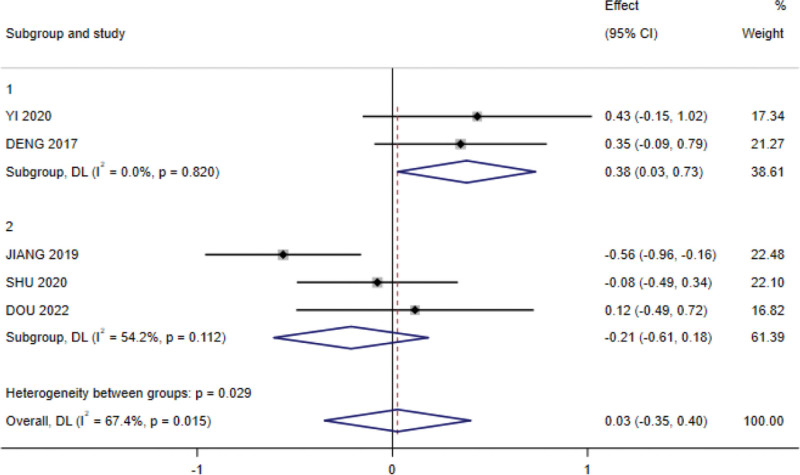
Forest plot for subgroup analysis of the effect of radiotherapy dose on BMI. BMI = body mass index.

In the subgroup analysis examining the effect of radiotherapy dose on BMI, 2 RCTs were included in the ≥ 70 Gy subgroup and 3 RCTs in the < 70 Gy subgroup. The analysis showed no heterogeneity in the ≥ 70 Gy subgroup (I^2^ = 0.00%; *P* = .82), with significant results (SMD = 0.38, 95% CI: 0.027–0.73; *P* < .05); the subgroup of < 70 Gy had no effect on BMI and showed heterogeneity between studies (I^2^ = 54.2%; *P* < .05), with a significant difference (SMD=−0.212, 95% CI: −0.607 to 0.184; *P* < .05; Fig. 10).

### 4.3. Publication bias analysis

Risk of bias analysis was performed only for 9 or more RCTs that described albumin levels as an indicator, and the results showed an asymmetric albumin funnel plot with Egger test *P* = .019, indicating a possible risk of publication bias (Supplemental Digital content Figure S1, http://links.lww.com/MD/K109). Furthermore, the cut-and-patch method was performed, and the results were stable before and after the cut-and-patch method; publication bias had only less effect on the authenticity of the results (We did not assess risk of publication bias because of the limited number of RCTs assessed [less than 9] in other analyses).

### 4.4. Sensitivity analysis

Sensitivity analyses were conducted using the study-by-study approach. The results of the analyses of the indicators BMI, albumin, prealbumin, and hemoglobin showed no significant change in the heterogeneity between studies, indicating a degree of reliability, possibly suggesting that the influencing factors in this meta-analysis are stable (Supplemental Digital content Figure S2, http://links.lww.com/MD/K110).

## 5. Discussion

Previous studies have reported that serum nutritional indicators can be used as predictors of a patient's nutritional risk in NPC, the presence of which is closely related to the patient prognosis.^[12,33,34]^ Radiotherapy is effective for patients with NPC when compared with chemotherapy alone, but the treatment can lead to adverse effects.^[35,36]^ The occurrence of adverse effects such as oral mucositis in patients with NPC can lead to a reduction in the intake and absorption of nutrients, resulting in further deterioration of the nutritional status of patients with NPC. The risk of malnutrition experienced by patients with NPC during radiotherapy will affect clinical outcomes and prognosis, thereby reducing patients’ quality of life. For cancer patients, early and aggressive enteral nutritional support therapy and oral nutritional supplementation, nasal feeding or parenteral intravenous nutrition therapy if necessary; With regard to parenteral nutrients, omega-3 proved its beneficial effect on serum lipid profile and oxidative stress, and glutamine can improve tolerance to radiotherapy and enhance the efficacy of radiotherapy in controlling tumor cells in oncology patients.^[1,37]^

Our systematic review included 10 clinical RCTs, and the analysis results showed that the nutritional indicators prealbumin, hemoglobin, and lymphocyte count significantly improved in patients receiving nutritional support during radiotherapy for NPC compared with those of control subjects, and the incidence of oral mucositis was significantly reduced when compared with those of control subjects.

Nutritional interventions during radiotherapy in patients with cancer are beneficial in improving the serum nutritional indicators and reducing the toxic effects of chemoradiotherapy, which is consistent with the results of a previous systematic evaluation.^[38]^ Related studies have shown that BMI, hemoglobin, total protein, albumin, and prealbumin are important indicators for evaluating malnutrition^[39]^; and that changes in BMI are significant during synchronous radiotherapy in patients with cancer; that improving and maintaining body weight is an important requisite for ensuring efficacy; and that BMI is also an independent prognostic factor for survival in NPC.^[40,41]^ The current study showed no significant difference in the change in BMI before and after the nutritional intervention during chemoradiotherapy, which is consistent with the findings of Bullock study,^[34]^ wherein the author indicated no correlation between BMI and patient prognosis, a result that needs to be validated by expanding the sample size. Our results indicate that no significant difference was obtained in albumin and total protein levels before and after the nutritional intervention. This result may be due to the small sample size and large error, which may have led to unstable results, and this finding is contrary to the results of a Chinese meta-analysis^[42]^; therefore, we believe that the effect of nutritional intervention on albumin and total protein levels is controversial, and serum albumin levels are an indicator of the nutritional status of the body. However, the current RCTs have not directly confirmed that nutritional interventions improve this indicator. Indeed, albumin does not appear to reliably indicate short-term nutritional changes in patients with cancer during radiotherapy because of the long half-life and the various disease processes that can influence albumin levels.^[43]^ As for radiotherapy toxicities, the incidence of oropharyngeal mucosal damage was significantly better in the nutritional intervention group than in the control group. Mucosal damage occurs in addition to malnutrition.^[44]^ Oropharyngeal mucosal damage not only reduces the number of nutrients consumed by patients with NPC and the tolerance of the body but also affects the efficacy of radiation therapy.

In this study, further subgroup analysis was performed on the radiotherapy dose and the nutritional intervention, and subgroup analysis of radiotherapy dose was referred to the radiation dose guidelines for NPC.^[45]^ The results of the subgroup analysis showed that albumin levels and BMI in the ≥ 70 Gy dose subgroup were significant after the nutritional intervention, and the reason may be attributed to dose toxicity. The albumin levels were higher and BMI improved significantly when compared with those in the control group. Previous research said radiation dose is associated with the incidence of adverse reactions and increased weight loss, weight loss often leads to the occurrence of malnutrition, the amount of radiation dose produced affects its toxicity, and the radiation dose will affect the effect of nutritional intervention.^[46]^ No significant differences were observed between the nutritional intervention subgroups for both albumin and BMI, possibly because of poor compliance with treatment in both cases, a finding that was consistent with those of Yamamoto et al.^[47]^ Oral nutritional supplements have good compliance and are the preferred nutritional therapy method for radiotherapy patients. When oral nutritional supplements cannot meet the target requirements or patients cannot eat orally, enteral nutrition tube feeding should be selected. The way of nutritional intervention will affect the patient compliance and thus the intervention effect. In addition, most RCTs had a high-quality bias because of the presence of individualization in the nutritional interventions received and the inability of RCT researchers to communicate directly with the participants, which may have affected some estimates of the effects of nutritional interventions. Further high-quality, large RCTs of these studies are needed to improve the homogeneity between intervention types. Two studies described the BMI group in the individualized enteral subgroup analysis in this meta-analysis, with a small number of cases of outcome events and significant inter-study heterogeneity; therefore, further additional studies are still needed.

As shown by the funnel plot, there may have been some publication bias in albumin levels in this study, which may be due to the small number of English-language publications included in the meta-analysis; articles with positive results, compared with negative results, being more likely to be published; and the fact that some RCTs did not have detailed data.

Several limitations of this study include the small sample size and the average quality of the literature and the fact that the regions studied were all from China, thus limiting the generalizability of the results obtained from this study; the difficulty of allocation concealment and blinded implementation attributed to the methodology of nutritional support for clinical events; the lack of specific data on other indicators such as liver and renal function (bilirubin and transaminases) and prognostic nutritional indices, which did not allow for systematic analysis; and the number of certain indicators included in the literature The heterogeneity among studies may be influenced by factors such as the timing of nutritional interventions and radiotherapy regimens for patients with NPC. Therefore, there is an urgent need for more clinical RCTs with large sample sizes, multiple assessment indicators, and long intervention and follow-up durations to assess the effectiveness of nutrition studies in patients undergoing radiotherapy for NPC. At present, there is no clinical study report on the effect of nutritional intervention on the increased recurrence rate of tumor metastasis. Whether it is for patients with new cancer or patients with recurrent cancer, nutritional intervention is necessary at the same time as radiotherapy, especially for patients with intake and absorption disorders, which can ensure the completion of radiotherapy program.

In conclusion, this meta-analysis found that nutritional interventions can improve the nutritional status of patients with NPC during chemoradiotherapy, and the results contribute to the clinical outcome of patients with NPC. Individualized nutritional support is a method that should not be overlooked to improve the nutritional status of patients undergoing concurrent chemoradiotherapy for NPC. The choice of nutritional support modality, the composition of nutritional elements, and the selection of nutritional agents should be based on the actual condition of the patients. Most of the current RCTs on nutritional support and NPC have not documented and performed a follow-up of the results of pre-nutritional risk screening and nutritional status of subjects after the termination of chemoradiotherapy and lack reliable nutritional parameters. Further designed studies with larger sample sizes and more standardized studies are needed to assess the effect of nutritional support strategies on radiotherapy for NPC. A report released by the World Health Organization in 2019 pointed out that more nutrition interventions should be included in the basic medical service system, increase rational investment in the field of nutrition, and improve the nutritional level of a person at all stages of life, and nutrition intervention should become one of the important components of national basic medical services.

## Acknowledgments

We sincerely thank all authors and study participants for their support in the study.

## Author contributions

**Conceptualization:** Tong Yang.

**Data curation:** Ying He, Xiaoxia Liu, Tong Yang.

**Formal analysis:** Ying He, Tong Yang.

**Investigation:** Sitao Tan.

**Methodology:** Ying He.

**Project administration:** Yan Li, Sitao Tan.

**Resources:** Xiaoxia Liu, Yan Li.

**Supervision:** Ying He.

**Validation:** Ying He.

**Software:** Yan Li.

**Writing – original draft:** Ying He.

**Writing – review & editing:** Xiaoyu Chen.

## Supplementary Material
