# Supplementary material for: Nutritional intervention for the prognosis of nasopharyngeal carcinoma chemoradiotherapy patients: A meta-analysis
Source: Medicine (Baltimore). 2023 Oct 13;102(41):e35386. doi: 10.1097/MD.0000000000035386 (PMC10578778; doi:10.1097/MD.0000000000035386)
Supplement: Supplementary file 1 [file medi-102-e35386-s001.docx]

**Table S1**. Specific search strategies.

| **Database Specific search strategy** | | **Number of hit** |
| --- | --- | --- |
| pubmed | (((((((((nutrition[MeSH Terms]) OR (nutrition support[Title/Abstract])) OR (Nutrition Therapy[Title/Abstract])) OR (enteral[Title/Abstract])) OR (Parenteral[Title/Abstract])) OR (fatty[Title/Abstract])) OR (amino acid[Title/Abstract])) AND (((((((((nasopharyngeal carcinoma[MeSH Terms]) OR (nasopharynx carcinoma[Title/Abstract])) OR (nasopharyngeal cancer[Title/Abstract])) OR (nasopharynx cancer[Title/Abstract])) OR (nasopharyngeal neoplasms[Title/Abstract])) OR (nasopharynx neoplasms[Title/Abstract])) OR (nasopharyngeal tumor[Title/Abstract])) OR (nasopharynx tumor[Title/Abstract])) OR (NPC[Title/Abstract])) | 412 |
|  | ((((((randomized controlled trial[Publication Type]) OR (controlled clinical trial[Publication Type])) OR(randomized[Title/Abstract])) OR (placebo[Title/Abstract])) OR (clinical trials as topic[Title/Abstract])) OR (randomly[Title/Abstract])) OR (trial[Title/Abstract]) |  |
| Cochrane | (nutrition:ab,ti OR 'nutrition support':ab,ti OR 'nutrition therapy':ab,ti OR enteral:ab,ti OR parenteral:ab,ti OR tnf:ab,ti OR 'famino acid':ab,ti OR fatty:ab,ti OR tna:ab,ti OR tpf:ab,ti) | 97 |
|  | ('nasopharyngeal carcinoma':ab,ti OR 'nasopharynx carcinoma':ab,ti OR 'nasopharyngeal cancer':ab,ti OR 'nasopharynx cancer':ab,ti OR 'nasopharyngeal neoplasms':ab,ti OR 'nasopharynx neoplasms':ab,ti OR 'nasopharyngeal tumor':ab,ti OR 'nasopharynx tumor':ab,ti OR npc:ab,ti) AND ([cochrane review]/lim OR [controlled clinical trial]/lim OR [systematic review]/lim OR [randomized controlled trial]/lim) |  |
| Embase | nutrition'/exp OR nutrition support OR nutrition therapy OR enteral OR parenteral | 65 |
|  | nasopharyngeal carcinoma'/exp OR nasopharynx carcinoma OR nasopharyngeal cancer OR nasopharynx cancer OR nasopharyngeal neoplasms OR nasopharynx neoplasms OR nasopharyngeal tumor OR nasopharynx tumor |  |
|  | randomized controlled trial':it OR 'randomized controlled trial'/exp OR 'randomized controlled trial' OR (randomized AND controlled AND ('trial'/exp OR trial)) OR 'crossover procedure'/exp OR 'crossover procedure' OR 'double blind procedure'/exp OR 'double blind procedure' OR '(randomi$ed':ti,ab,kw OR 'randomly)':ti,ab,kw OR '(crossover$':ti,ab,kw OR 'cross-over$)':ti,ab,kw OR 'placebo':ab OR '(doubl$ adj blind$)':ti,ab,kw OR 'assign$':ab OR 'allocat$':ab |  |
| CNKI | (SU='yingyang') AND (SU % 'biyanai') AND (SU % 'changneiyingyang'+'changwaiyingyang') | 12 |
| VIP | (U=yingyang OR U=changwai OR U=changnei) AND (U=biyanai) | 10 |
| Wanfang | Title/Abstract/Keyword:(yingyang) AND Title/Abstract/Keyword:(biyanai) | 11 |
