# Supplementary figures and images for: Nutritional intervention for the prognosis of nasopharyngeal carcinoma chemoradiotherapy patients: A meta-analysis
Source: Medicine (Baltimore). 2023 Oct 13;102(41):e35386. doi: 10.1097/MD.0000000000035386 (PMC10578778; doi:10.1097/MD.0000000000035386)

**Figure S1** Albumin indicator funnel chart


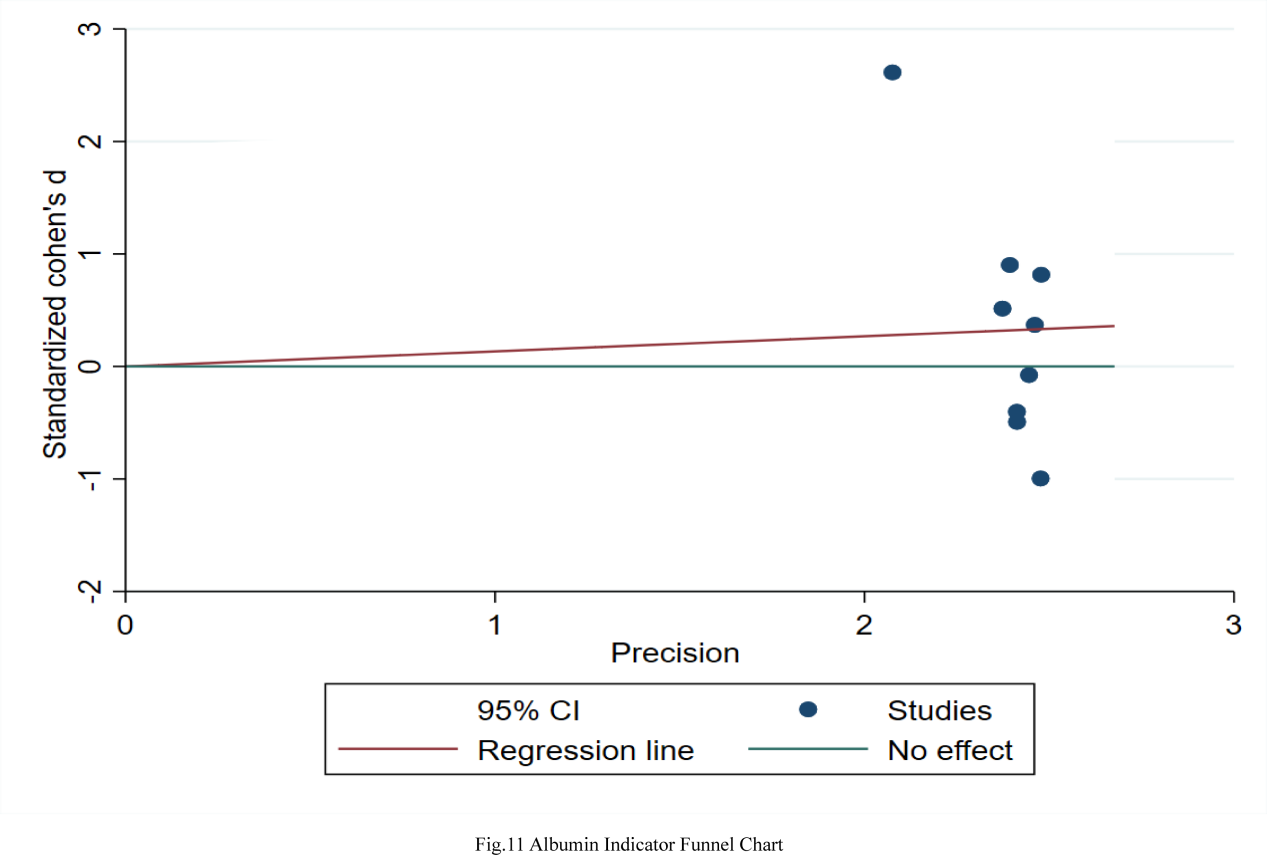

Supplement: Supplementary file 2 [file medi-102-e35386-s002.docx]
