# Supplementary material for: Nutritional intervention for the prognosis of nasopharyngeal carcinoma chemoradiotherapy patients: A meta-analysis
Source: Medicine (Baltimore). 2023 Oct 13;102(41):e35386. doi: 10.1097/MD.0000000000035386 (PMC10578778; doi:10.1097/MD.0000000000035386)
Supplement: Supplementary file 3 [file medi-102-e35386-s003.docx]

**Figure S2** Sensitive analysis:

1. BMI

1. albumin

1. pre-albumin

d) hemoglobin
